# Homogeneity Guarantee of Nickel Reference Material in Soybean Matrix: Influence Mechanism of Particle Size Distribution

**DOI:** 10.3390/foods15091513

**Published:** 2026-04-27

**Authors:** Nuojia Wang, Zengwang Guo, Yanxiang Wu, Jin Ye, Lin Zhu, Yue Wang, Zhongjiang Wang, Songxue Wang, Minghui Zhou

**Affiliations:** 1 College of Food Science, Northeast Agricultural University, Harbin 150030, China; 15231635382@163.com (N.W.); gzwname@163.com (Z.G.); 2 NFSRA Key Laboratory of Grain and Oil Quality and Safety, Academy of National Food and Strategic Reserves Administration, Beijing 100037, China; wyx@ags.ac.cn (Y.W.); yj@ags.ac.cn (J.Y.); zl@ags.ac.cn (L.Z.); wy@ags.ac.cn (Y.W.); zmh@ags.ac.cn (M.Z.)

**Keywords:** soybean, reference material, homogeneity, influence mechanism, span

## Abstract

In response to the demand for reference material under the EU Maximum Levels for Nickel (Ni) limit in soybeans (15 mg/kg) in 2024, this study explored the technical difficulty of ensuring the homogeneity of Ni reference material in the soybean matrix. Multi-scale characterization (LA-ICP-MS, ICP-MS, FT-IR, etc.) verified that Ni was specifically enriched in embryo and the finer powder (mainly embryo). Based on this finding, we innovatively proposed the span [(D_90_ − D_10_)/D_50_] as a rapid predictor to evaluate homogeneity, offering a potential screening tool to optimize grinding conditions and reduce reliance on time-consuming traditional homogeneity assessments (Ni-RSD by ICP-MS). A positive correlation between span and homogeneity was observed, which was attributed to the inhomogeneous distribution of low-Ni tissue (seed coat). By optimizing the crushing process (hammer cyclone milling, room temperature: 20 °C, 15,000 r/min, ≤ 0.45 mm sieve), a homogeneity uncertainty of 1.00% was obtained. This finding helps in ensuring the homogeneity of reference materials from other high-fat oilseed matrixes.

## 1. Introduction

As an important agricultural product in the world, soybeans are rich in various nutrients, containing about 40% protein, 25% carbohydrates, 20% oil, 10% cellulose, 4% minerals, and a range of bioactive components. This nutritional composition makes soybean a key source of high-quality plant protein for human consumption and an essential raw material in animal feed [1]. However, in addition to providing essential nutrients, soybeans also pose potential food safety risks—Ni pollution has been one of them. Ni is not only an essential trace element for soybean but also a confirmed human carcinogen, associated with metabolic disorders, antibiotic resistance, intellectual disability, neurodegenerative diseases, and other health issues [2]. More critically, the International Agency for Research on Cancer (IARC) has classified nickel compounds as Group I carcinogens [3]. In view of the potential health threat of Ni, the European Commission issued Regulation (EU) 2024/1987 in July 2024, amending the maximum residue limit of Ni in soybeans to 15 mg/kg [4]. The implementation of this stricter limit demands greater reliability and accuracy in testing data. In order to ensure the accuracy and comparability of test data, certified reference materials are essential quality control tools. However, compared with other grains, the soybean matrix contains higher levels of oil, protein, and other components, posing greater challenges to analytical accuracy during detection. The use of matrix-matched reference materials helps address this issue by reflecting the true speciation and interaction of contaminants within the complex matrix, thereby effectively correcting matrix effects during analysis [5]. However, there are few types of existing soybean matrix reference materials. In routine testing, pure standard solutions are often used for calibration due to their low cost and convenience. However, they lack matrix matching and cannot account for sample preparation losses or matrix effects, making them potentially unreliable for accurate quantification [6]. Reference materials, which are matrix-matched and certified, can overcome these limitations [7], but they are not routinely used in high-throughput testing because of high cost, limited availability, and greater consumption. Instead, reference materials are typically reserved for method validation, inter-laboratory comparisons, and periodic accuracy checks [8].

Compared with other grain samples, the development of reference material for Ni analysis in soybean has proceeded more slowly. On the one hand, Ni is an essential micronutrient for plants, and distinct biological activities of the seed coat and embryo in soybean seeds result in an inherent spatial gradient in the natural distribution of Ni [9]. This biologically driven variability means that even thoroughly cleaned and well-processed soybeans may exhibit significant Ni heterogeneity—a factor that is less critical for non-essential elements. Therefore, understanding the tissue-specific distribution of Ni is not merely an analytical concern but a prerequisite for developing a fit-for-purpose Ni certified reference material (CRM). On the other hand, the soybean matrix is compositionally complex. The embryo is rich in protein and oil, whereas the seed coat consists mainly of cellulose. These compositional differences confer distinct physical properties: the embryo is relatively friable, while the seed coat is harder and tougher [10]. As a result, during milling, the embryo is easily ground into fine particles, whereas the seed coat tends to remain as coarse fragments. This differential grinding behavior leads to a bimodal particle size distribution, further contributing to the inhomogeneous distribution of Ni in the subsequent development of reference materials. These challenges impose higher demands on the representativeness of trace samples used in heavy metal detection and pose greater difficulties in ensuring the homogeneity of reference materials with a soybean matrix.

Based on the structural characteristics of soybean seeds, it can be inferred that the key to ensuring homogeneity lies in controlling the relationship between composition and particle size; in other words, ensuring that both the embryo and seed coat can be ground to similar particle sizes. Historically, studies on homogeneity assurance in similar matrices have consistently regarded particle size distribution as a critical influencing factor, often leading to an excessive pursuit of powder fineness [11]. However, in oil-rich soybeans [12], traditional grinding methods tend to release more oil and protein, ultimately resulting in particle segregation [13]. Therefore, it is necessary to systematically investigate the distribution patterns of Ni and the crushing characteristics of the soybean matrix. By precisely regulating the preparation parameters, we can control the physical state changes in the material before and after crushing, thereby minimizing extreme particle size differentiation in soybean powder and ensuring the homogeneity of the resulting reference material.

To address these challenges, this study developed a multi-scale collaborative strategy to ensure homogeneity throughout the sample preparation process. Based on the understanding of the initial distribution of Ni in soybean tissues, we compared cryogenic and ambient-temperature grinding to maximize homogeneity. Low-temperature treatment can make the sample brittle, prevent agglomeration, and improve homogeneity. In addition, the effects of rotational speed and powder particle size on homogeneity were compared [14]. A multi-dimensional evaluation system was subsequently established, integrating overall particle size distribution analysis with fraction-specific Ni quantification to characterize and verify homogeneity. This approach enabled elucidation of the underlying mechanism by which particle size distribution governs Ni inhomogeneity. Collectively, the findings establish a comprehensive parameter framework to guide homogeneity guarantee and offer a transferable methodology for ensuring elemental homogeneity in other high-fat oilseed matrices.

## 2. Materials and Methods

### 2.1. Materials and Instruments

Soybeans used in this study were purchased from Chaoyang City, Liaoning Province, China. All chemical reagents were of analytical grade, and laboratory water was prepared using a Milli-Q water purification system (MilliporeSigma, Burlington, MA, USA). Hammer cyclone milling (Model M125C2, Pufeng Hengxin, Guangzhou, China) features a built-in temperature protection circuit and high-speed airflow for heat dissipation, keeping the chamber temperature within the set limit.

### 2.2. Sample Powder Preparation

For each milling condition, 1 kg of soybeans was processed. After the initial grinding, the material was sieved using a 0.45 mm test sieve for final sample preparation. Separately, a portion of the coarsely ground powder was sieved through a series of screens (including 0.90 mm) to assess nickel distribution across particle sizes. The fraction retained on the sieve was returned to the mill for additional grinding. In general, this cycle was repeated an average of 2 times to allow the entire sample (40 mesh, 0.45 mm) to pass through the sieve. The combined powder that passed the sieve was collected, homogenized by gentle mixing, and used for subsequent analyses.

### 2.3. LA-ICP-MS Measurement

The distribution of Ni in soybean seeds was measured according to the method described by Zhang et al. [15]. Dried grains were fixed with AB glue, and longitudinal sections (along the crease) were prepared using a paraffin sectioning machine (Model SM2010R, Leica Biosystems, Shanghai, China). The longitudinal sections were then analyzed using a femtosecond laser ablation system (fsLA, Teledyne Photon Machines, Bozeman, MT, USA) coupled with inductively coupled plasma time-of-flight mass spectrometry (ICP-TOF-MS, Shanghai, China). A 343 nm laser was operated at an ablation frequency of 50 Hz, with a square beam size of 15 μm × 15 μm, laser energy set to 100%, and a scanning speed of 750 μm/s. The ablated aerosols were transported to the dry plasma of the ICP-MS using a mixture of helium and hydrogen as carrier gas at a flow rate of 1.3 L/min [16]. For quantitative analysis, a calibration curve was established using a Ni standard solution. A series of concentration gradients (0, 0.5, 1, 10, 50, 100 mg/kg) were prepared, and under the same laser ablation and mass spectrometry acquisition conditions, the signal intensity (CPS) corresponding to each concentration was measured. A linear calibration curve (R^2^ > 0.99) was obtained by fitting.

### 2.4. ICP-MS Measurement

Accurately weigh 0.2 g of soybean samples (accurate to 0.0001 g) and transfer them into digestion vessels. Add 6 mL of nitric acid and seal the vessels tightly. The samples were then digested using a microwave digestion system under the conditions specified in Table 1. After the digestion vessels had cooled, the contents were quantitatively transferred and made up to 50 mL for analysis. The operating parameters for ICP-MS measurement are shown in Table 2 [17].

As shown in Figure S1, Tables S1–S5, after systematic methodological investigation, Specifically, method and instrument repeatability both gave RSD ≤ 1.64% (*n* = 7); the LOD was 1.55 × 10^−5^ mg/kg (3σ/k) and the LOQ was 4.64 × 10^−5^ mg/kg (3 × LOD); recoveries at three spiked levels ranged from 95.72% to 101.20%; and the matrix effect, assessed by the slope ratio (matrix/solvent = 1.0085), fell within the acceptable range of 0.8–1.2, indicating negligible matrix effect. These validation results confirm the reliability of the ICP-MS measurements.

### 2.5. Ni-RSD

To evaluate the homogeneity of the Ni mass fraction in soybean powder, 3 bottles (units) were randomly selected from each grinding condition. From each bottle, 3 sub-samples were weighed and analyzed by ICP-MS. The relative standard deviation of Ni(Ni-RSD) was calculated based on independent measurements.

### 2.6. Particle Size Distribution

The particle size distribution of the soybean powder was determined following the method of Zhang et al. [18], using a laser diffraction particle size analyzer (PSA 1190, Anton Paar GmbH, Graz, Austria) with a dry dispersion unit. Operating in dry dispersion mode, the sample was dispersed with an air pressure of 2500 to 3000 bar, and the feed rate was set above 40% to ensure complete dispersion, with an obscuration rate between 0.5% and 7%. All measurements were performed at 25 ± 1 °C. The particle size distribution was characterized by the D_10_, D_50_, and D_90_ values, with each sample analyzed in triplicate and the average reported.

The definition of span is as follows:

(1) $$span=\frac{D_{90}-D_{10}}{D_{50}}$$

Among them, D_10_, D_50_, and D_90_ represent the particle size diameters at which the cumulative volume of particles reaches 10%, 50%, and 90% of the total, respectively. A smaller span indicates a more concentrated particle size distribution, whereas a larger span reflects a more dispersed distribution [19].

### 2.7. Extraction of Surface Free Fat

Surface free fat is the fat covering the surface of the soybean powder granules that is not encapsulated inside the soybean powder granules, which is easily eluted by organic reagents. According to the method described by Zhou et al. [20], 2.00 ± 0.01 g of soybean powder sample was placed on filter paper in a glass funnel and washed four times with 5 mL hexane at 30 s intervals. The filtrate was collected in a pre-dried, constant-weight aluminum dish, and the sample was subsequently dried in an electric thermostatic oven at 80 °C to a constant weight. The surface free fat was calculated by Equation (2).

(2) $$Surface=\frac{M_{1+2}-M_{1}}{M_{3}}\;$$

where M_1+2_ is the total weight of the aluminum dish and surface fat (g), M_1_ is the weight of the aluminum dish (g), M_3_ is the weight of the soybean powder sample (g).

### 2.8. Characterization of Powder Physical Properties

To comprehensively evaluate the suitability of the milled powders as candidate reference materials, the physical properties—including bulk density, tapped density, and flowability (expressed as the Carr index, CI)—were determined for all produced powders according to the method described by Yikilkan et al. [21]. These physical parameters are critical for CRM production: good flowability ensures consistent bottle filling and minimizes weight variation among units, while bulk and tapped density data inform packaging design. Correlating these physical properties with milling conditions provides a more complete understanding of how processing parameters influence the end-use performance of candidate reference materials.

#### 2.8.1. Bulk Density

A measuring cylinder was filled with 100 mL of powder, and its weight was recorded. Bulk density was calculated as the powder weight (g) divided by the powder volume (100 mL). This parameter reflects the compactness of the powder in its natural, loose state. Under the fixed volume, the loose-filling method used in this study; a lower bulk density indicates larger interparticle voids, which may suggest more irregular or finer particles [22].

#### 2.8.2. Tapped Density

The sample for tapped density measurement was added to a 100 mL top-fitted scale cylinder and knocked 100 times to remove the gap between the particles. Add more powder to the gauge and tap again until it reaches a steady state. The calculation formula is the weight of the compacted powder (g) divided by the powder volume (100 mL). It reflects the maximum bulk density of the powder after vibration or compaction. The difference between value and bulk density can reflect the compressibility of the powder.

#### 2.8.3. Carr Index (CI)

The value of Carr index is the ratio of the difference between tapped density and bulk density to tapped density. Flowability is positively correlated with the Carr index value; a lower CI indicates better flowability, while a higher CI suggests poorer flowability.

### 2.9. FT-IR

Referring to the practice of Zhang et al. [23], infrared spectra of freeze-dried powder were recorded between 4000 and 400 cm^−1^ using an FT-IR spectrophotometer. Before the test, lyophilization samples and potassium bromide (KBr) were combined in a 1:100 ratio and pressed into disks. The average number of scans per sample was 14, at 4 cm^−1^ resolution.

### 2.10. Determination by Scanning Electron Microscope

Referring to the practice of Zhang et al. [24], freeze-dried soybean powder samples were placed on a conductive silica gel strip. Use double-sided adhesive on the circular sample plate. After spraying gold, the specific morphology of the sample under SEM was observed at an accelerating voltage of 5.0 kV and an appropriate magnification.

### 2.11. Homogeneity Study

The homogeneity of the candidate reference material was assessed following the methodological framework described by Azevedo et al. [25]. The entire batch of soybean powder was divided into 80 bottles, each containing 10 g of material. For the homogeneity assessment, bottles were randomly selected according to statistical sampling principles.

#### 2.11.1. Between-Bottle Homogeneity

To evaluate between-bottle homogeneity, 8 bottles were randomly selected from the entire batch. From each bottle, a single test portion of 200 mg was subjected to three sampling tests (top, middle, and bottom) and analyzed by ICP-MS, and the experiment was repeated 3 times.

#### 2.11.2. Within-Bottle Homogeneity

To assess within-bottle homogeneity, three bottles were selected. From each bottle, ten test portions of 200 mg were taken from different positions (top, middle, and bottom) and analyzed separately, and the experiment was repeated 3 times.

One-way ANOVA was used to evaluate whether the between-bottle variance significantly exceeded the measurement variance. In addition, taking into account the recommendations of Azevedo et al. [25], the inherent uncertainty in the measurement deviation of the homogeneity test between bottles was calculated. Thus, the uncertainty due to the dispersion between the bottles (u_bb_) can be calculated by Equations (3) or (4).

(3) $$u_{bb}=\sqrt{\frac{\left({MS}_{between}-{MS}_{within}\right)}{n_{0}}}$$

(4) $$u_{bb}=\sqrt{\frac{{MS}_{within}}{n}}\cdot \sqrt[{4}]{\frac{2}{{vMS}_{within}}}$$

where MS_between_ is the mean square error (ANOVA) between the CRM batch units, MSwithin is the mean square error (ANOVA) of CRM, vMS_within_ is the degree of freedom of MS_within_, n or n_0_ denotes the number of measurements in the study. At the time where MS_between_ is greater than MS_within_, both equations are applicable, and the higher ubb value is selected. Conversely, if MSbetween is less than MS_within_, then only Equation (4) can be used.

### 2.12. Statistical Analysis

The experiments were performed in triplicate, and results are represented as mean values ± standard deviations (SD). Duncan’s test was used after a one-way analysis of variance to compare means, with *p* < 0.05 indicating statistical significance.

## 3. Results

### 3.1. Study on the Distribution of Ni in Soybean

#### 3.1.1. Distribution of Ni in Soybean

**Figure 1:**
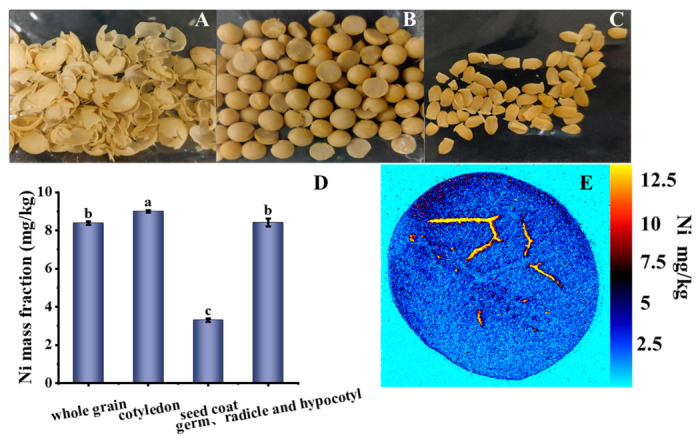
Ni distribution and mass fraction in soybean seed components. (**A**) Seed coat, (**B**) cotyledon, (**C**) embryo (including germ, hypocotyl, and radicle), (**D**) quantification of Ni mass fraction in different seed components, (**E**) LA-ICP-MS imaging of Ni distribution in a longitudinal section of a soybean seed. Different lowercase letters indicate significant differences (*p* < 0.05).

As shown in Figure 1A–C, the soybean grain could be separated into three parts: seed coat; cotyledon; germ, radicle, and hypocotyl, with the last four constituting the embryo. The specific weight of each part is presented in Table 3. The mass fraction of the embryo (including cotyledon, germ, radicle, and hypocotyl) in the whole soybean was found to be approximately 91%. Ni mass fraction, in the whole grain; cotyledon; seed coat; germ, radicle, and hypocotyl, ranged from 3.29 to 8.99 mg/kg (Figure 1D). In soybean, the Ni concentration in cotyledon and germ, radicle, and hypocotyl was significantly higher than in seed coat (*p* < 0.05), which aligns with previous studies [26]. The Ni distribution in the soybean is important because it influences the homogeneity of Ni in the powder. The distribution of Ni was investigated in longitudinal sections of the soybean using the LA-ICP- MS technique (Figure 1E). A higher Ni signal intensity was clearly observed in this mapping, which was consistent with the Ni mass fraction results (Figure 1D). This phenomenon stems from the physiological function of Ni as a nutrient element [27]: it is a key component of the urease active center, and urease plays a central role in soybean nitrogen metabolism [28]. Therefore, Ni is physiologically preferentially allocated to metabolically active tissues (embryos) during seed germination and growth.

Finally, the physiological enrichment of Ni in metabolically active tissues of soybean grains presents a core challenge that is difficult to reconcile with the homogeneity required for the preparation of reference materials. Although this issue can be solved by using larger sample intakes, it becomes critical in CRM production, where sample sizes are necessarily limited. Understanding the sources of this heterogeneity—as we have done in this study—is therefore essential for developing processing strategies that improve homogeneity at a scale relevant to end users.

#### 3.1.2. Distribution of Ni in Powder

In order to systematically evaluate the homogeneity of the powder, this study used a combination of physical screening and chemical analysis. The soybean powder was graded using a series of standard test sieves with mesh sizes of 20 mesh (0.90 mm), 40 mesh (0.45 mm), 60 mesh (0.30 mm), 100 mesh (0.15 mm), and 120 mesh (0.125 mm). Subsequently, the Ni mass fraction in each particle size fraction was accurately determined by ICP-MS.

**Figure 2:**
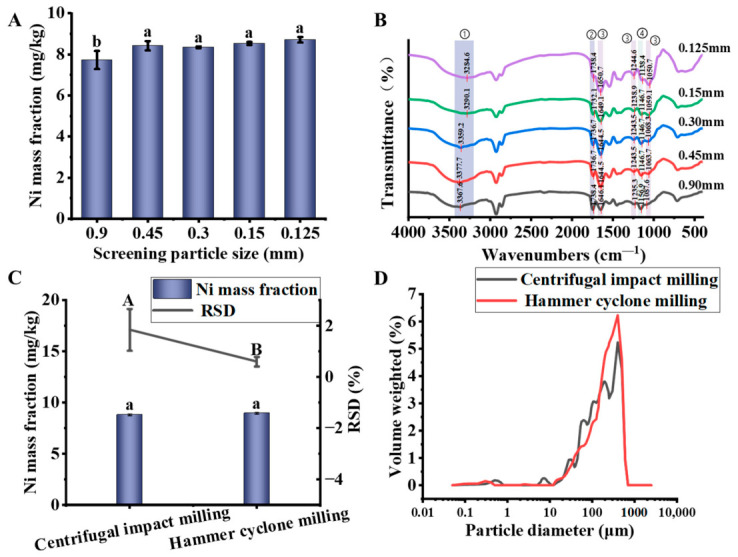
(**A**) Ni mass fraction in soybean powder of different sieve fractions, (**B**) FT−IR spectra of the corresponding sieve fractions, (**C**) Ni mass fraction and associated relative standard deviation (RSD) resulting from different millings, (**D**) particle size distribution characteristics resulting from different milling processes. Different lowercase letters indicate significant differences (*p* < 0.05). Different capital letters indicate significant differences (*p* < 0.05), which are distinguished from lowercase letters.

As shown in Figure 2A, screening the powder derived from the same soybean batch showed a systematic distribution of Ni across different particle size ranges. With decreasing particle size, the Ni mass fraction showed a gradual increase. Specifically, when the powder particle size was above 0.90 mm, the Ni mass fraction was 7.73 mg/kg, which was significantly lower than that in other particle sizes (*p* < 0.05). Based on the distribution of Ni in soybean tissues, this phenomenon can be attributed to the relatively large particle size of the seed coat after grinding. The seed coat is rich in cellulose, hemicellulose, and lignin, and its cell wall forms a highly ordered, cross-linked, multi-scale fibrous composite structure. This structure endows the seed coat with high toughness, tensile strength, and shear strength, leading predominantly to plastic deformation and tearing during conventional grinding processes [29]. As a result, it is difficult to achieve the desired fineness, and the seed coat tends to remain in the coarse particle fraction after grinding [30].

Consequently, the coarse fraction (>0.90 mm) was predominantly composed of seed coat material, as reflected by its low-Ni content—a direct consequence of the seed coat’s inherent elemental composition. This observation provides quantitative evidence of the physical segregation between seed coat and embryo during the milling process.

#### 3.1.3. FT-IR

The characteristic absorption peaks of different functional groups in the samples were identified by FT-IR, enabling effective discrimination of chemical composition differences and providing insights into the fragmentation behavior of different tissues during grinding [31].

Figure 2B shows the FT-IR spectra of powder fractions obtained after sieving the same soybean powder batch. The main absorption features are as follows: ① a broad absorption peak at 3000–3500 cm^−1^, corresponding to the -OH stretching vibrations of proteins or water; ② a stretching vibration of the ester carbonyl group (C = O) appears at 1730–1750 cm^−1^; ③ peaks at 1640–1660 cm^−1^ (N–H bending vibration of amino groups) and at 1244 cm^−1^ and 1063 cm^−1^ (C–N stretching vibrations), characteristic of proteins; and ④ C–O–C sugar ring vibrations from cellulose observed in the 1150–1000 cm^−1^ region [32]. In soybean seeds, the germ, hypocotyl, and radicle are metabolically active and rich in oil, while the seed coat possesses a robust structure composed primarily of cellulose and hemicellulose [33]. The cotyledons are responsible for nutrient storage and are rich in protein [34]. Therefore, these functional groups serve as effective markers for identifying proteins, polysaccharides, oils, and other components, allowing inference of the tissue origin of each particle size fraction.

According to the spectra, the ④ region (cellulose) exhibited a strong signal in the 0.9 mm fraction. This can be explained by the concentration of seed coat fragments in this coarse fraction. Concurrently, protein characteristic peaks were also observable in this fraction, likely due to the adherence or entrapment of proteinaceous tissue within or on the surface of larger seed coat fragments. As particle size decreased, the signal intensity of region ④ progressively weakened, while the signals of region ② (oil) and region ③ (protein) correspondingly increased. In the 0.125 mm fraction, region ② and region ③ dominated the spectrum, and region ④ signals were nearly absent, indicating that this finest fraction consisted almost entirely of embryo-derived material.

#### 3.1.4. Summary

This study demonstrates that particle size reduction alone is insufficient to guarantee homogeneity in soybean powder. Due to the contrasting physical properties and Ni distribution of the embryo and seed coat, excessive grinding can actually amplify tissue-specific segregation, resulting in greater compositional heterogeneity. This finding highlights the need for a mechanism-based approach to particle size control in reference material production. By integrating chemical analysis (Ni distribution) with physical characterization (particle size distribution, density, flowability), grinding conditions can be optimized to minimize segregation while achieving adequate fineness.

### 3.2. Selection of Crushing Tool

Due to the significant influence of the crushing tool on particle size distribution, an appropriate crushing tool was selected for further study. Currently, the commonly used crushing equipment includes centrifugal impact milling [35] and hammer cyclone milling [24]. Therefore, the effects of these two crushing tools were evaluated.

As shown in Figure 2C, the measured mass fractions of Ni were 8.82 mg/kg and 8.97 mg/kg. The Ni-RSD was used as the evaluation index. The Ni-RSD value obtained with the centrifugal impact mill was significantly higher (1.85%) than that with the hammer cyclone mill (0.60%) (*p* < 0.05). Centrifugal impact milling primarily relies on higher speed impact and rolling between the material and the crushing screen, which tends to produce particles with a wider size distribution during the crushing process [36]. This phenomenon can easily lead to particle size segregation, resulting in deviation during sampling. In contrast, the hammer cyclone milling combines the functions of hammer impact and airflow classification, resulting in a more concentrated particle size distribution in the powder. This improved sample homogeneity during the sampling process [18]. This conjecture was further verified by the particle size distribution results shown in Figure 2D and Table 4, which indicate that the particle size distribution of soybean powder prepared by the hammer cyclone milling was more concentrated, with a smaller span.

In addition, the processing efficiency of the centrifugal impact milling is limited due to its single injection volume and the need for frequent production interruptions for cleaning, leading to material waste and making it unsuitable for the continuous preparation of reference materials [37]. In contrast, the hammer cyclone milling offers a longer service life and higher crushing efficiency, making it more suitable for large-scale preparation of reference materials. Therefore, the hammer cyclone milling was selected for this study.

### 3.3. Study on Crushing Process Parameters

#### 3.3.1. Particle Size Control

Particle size was controlled in two stages: during milling by the built-in sieve of the hammer cyclone milling, and after milling by standard test sieves. This approach allowed the individual contributions of in-milling and post-milling sieving to Ni homogeneity to be assessed separately. The oversize fraction was re-ground until the entire sample passed the standard test sieve.

(1) Sieve size control

**Figure 3:**
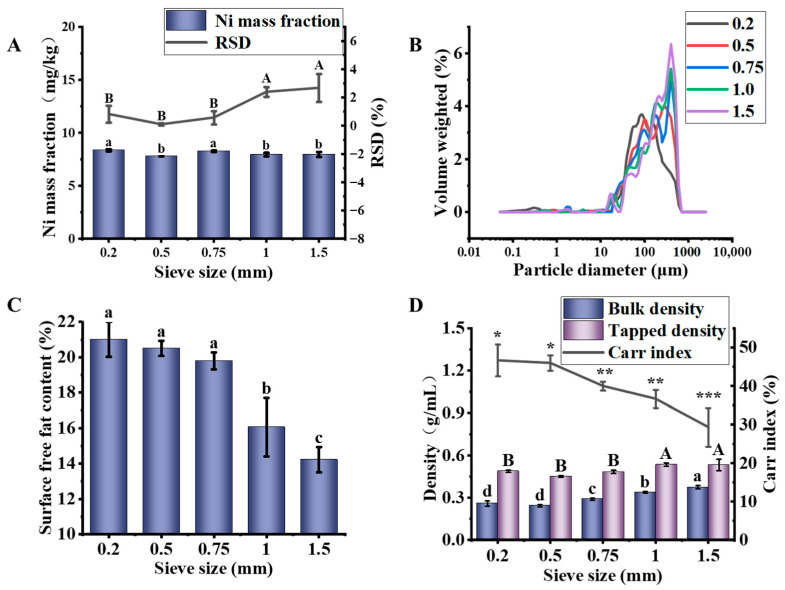
Effects of sieve size on the properties of soybean powder. (**A**) Ni mass fraction and its relative standard deviation (RSD), (**B**) particle size distribution, (**C**) surface free fat content, (**D**) particle differentiation characteristics. Different lowercase letters indicate significant differences (*p* < 0.05). Different capital letters indicate significant differences (*p* < 0.05), which are distinguished from lowercase letters. “*, **, ***” for Carr index serve the same purpose, indicating significant differences at *p* < 0.05, respectively, and are used only to visually separate this parameter from the density data.

As shown in Figure 3A, the Ni mass fraction ranged from 7.82 mg/kg to 8.38 mg/kg, but the samples exhibited significantly different homogeneity (*p* < 0.05). As the sieve size increased, the Ni-RSD showed a trend of decreasing first and then increasing, and reached an optimal value at a sieve size of 0.5 mm (Ni-RSD = 0.10%). This trend can be attributed to the fact that the 0.5 mm sieve represents an optimal balance. Finer sieves (<0.5 mm) might increase crushing heat and local adhesion, which could lead to particle segregation and, consequently, increased inhomogeneity [38]. Conversely, coarser sieves (>0.5 mm) might result in a wider span, making the powder more prone to segregation. This increased uncertainty during subsequent sampling operations, leading to another type of inhomogeneity [39].

As shown in Figure 3B and Table 5, as the sieve size increased, the span gradually decreased. This indicates that the particle size distribution of the powder became more concentrated and narrower after using a coarser sieve (1.0 or 1.5 mm). The fundamental reason for this phenomenon is that a larger sieve size allows coarser particles to pass through, while finer particles (those around 0.2 mm) are more likely to be retained. When using a smaller sieve (0.2 mm), strong shearing and repeated crushing generate a large number of finer particles in the final powder, resulting in a wider particle size distribution and an increased span value. This could lead to sampling bias and compromise homogeneity.

As shown in Figure 3C, as the sieve size increased, the surface free fat content decreased. The sample obtained with the 0.2 mm sieve exhibited the highest oil yield (21.02%), indicating that excessive crushing greatly increased the specific surface area and promoted the release of oil [40]. The oil yield of the sample obtained with the 1.5 mm sieve was the lowest (14.22%), but the standard deviation was larger than that of the other samples, further demonstrating that a wider particle size span can compromise homogeneity. This suggests that the control of sieve size not only affects the physical properties of the powder but also influences the chemical composition, thereby playing a key role in determining homogeneity.

As shown in Figure 3D, with increasing sieve size (coarser particles), both bulk density and tapped density of the soybean powder increased. This can be explained by the fixed-volume measurement method: fine powders with poor flowability bridge and pack poorly when poured loosely, giving lower mass per fixed volume; coarser particles flow better and settle more completely, resulting in higher bulk density. Concurrently, as sieve size increased, the Carr index significantly decreased from 46.67% to 29.33% (*p* < 0.05), indicating a significant improvement in flowability. This is consistent with the general principle that coarser particles flow better due to lower interparticle cohesion and reduced tendency to aggregate [41]. Although the coarser powder exhibited a lower Carr index (higher flowability), its larger standard deviation suggests that segregation may still occur during transportation and sampling, potentially leading to fluctuations in analytical results.

(2) Standard test sieve mesh

Based on the above results, the powders obtained using 0.45 mm (40 mesh) and 0.3 mm (60 mesh) sieves were considered relatively suitable, as they were neither excessively crushed nor insufficiently crushed. Therefore, this study compared standard test sieves of 0.45 mm and 0.3 mm to evaluate their effects on homogeneity.

**Figure 4:**
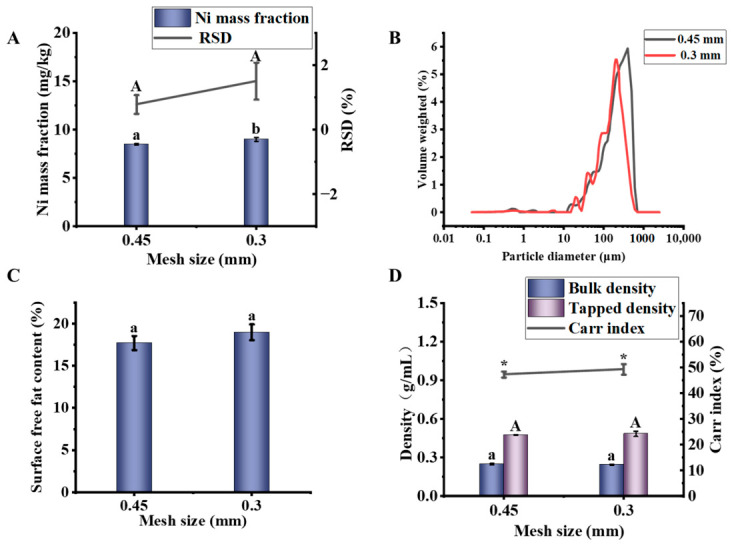
Effects of mesh number on the properties of soybean powder. (**A**) Ni mass fraction and its relative standard deviation (Ni-RSD). (**B**) Particle size distribution. (**C**) Surface free fat content. (**D**) Particle differentiation characteristics. Different lowercase letters indicate significant differences (*p* < 0.05). Different capital letters indicate significant differences (*p* < 0.05), which are distinguished from lowercase letters. “*” for Carr index serve the same purpose, indicating significant differences at *p* < 0.05, respectively, and are used only to visually separate this parameter from the density data.

As shown in Figure 4A, it was found that the Ni mass fraction at 0.3 mm (9.01 mg/kg) was significantly higher than that at 0.45 mm (8.50 mg/kg), while the Ni-RSD at 0.3 mm (1.51%) was also higher than that at 0.45 mm (0.78%). This may be attributed to the balance between particle size and physical stability: the powder obtained with the 0.45 mm sieve had a moderate particle size and a concentrated particle size distribution, which effectively minimized electrostatic adsorption effects commonly observed in fine powders [42], thereby resulting in better homogeneity in subsequent processes. In contrast, the powder obtained with the 0.3 mm sieve required multiple crushing steps, which could readily enhance electrostatic effects and lead to particle segregation.

As shown in Figure 4B and Table 6, although a finer average particle size was achieved using the 0.3 mm sieve, the span of the particle size distribution increased, indicating greater variability within the powder [43]. A wide particle size distribution is often the starting point of homogeneity deterioration, ultimately compromising the homogeneity of the powder.

As shown in Figure 4C, no significant difference was observed in the surface free oil content between powder fractions obtained using 0.3 mm and 0.45 mm mesh sieves (*p* > 0.05, *n* = 3). Error bars represent the standard deviation (SD) from three independent replicates. To ensure that all powder passed through the 0.3 mm sieve, some samples were subjected to repeated milling. Although this process resulted in a slightly higher surface oil content compared to the other group, the difference was not statistically significant (*p* > 0.05). This slight increase, though not significant, likely reflects particle size segregation caused by repeated milling (generation of finer particles with larger specific surface area), which can in turn amplify inhomogeneity across fractions.

As shown in Figure 4D, the bulk density, tapped density, and flowability of the different samples showed no significant differences (*p* > 0.05). However, the repeatability observed with a 0.3 mm sieve was poor, indicating that segregation may occur during the experiment.

In summary, the use of the 40 mesh (0.45 mm) standard test sieve effectively avoided negative factors such as electrostatic effects and ensured good homogeneity.

#### 3.3.2. Crushing Temperature

For high-fat and high-protein materials such as soybeans, temperature control has a significant effect on the crushing process. Lower temperatures increase oil viscosity, potentially leading to segregation and reduced homogeneity. Conversely, higher temperatures can cause protein denaturation and oil oxidation. Therefore, this study aimed to determine the optimal grinding temperature.

**Figure 5:**
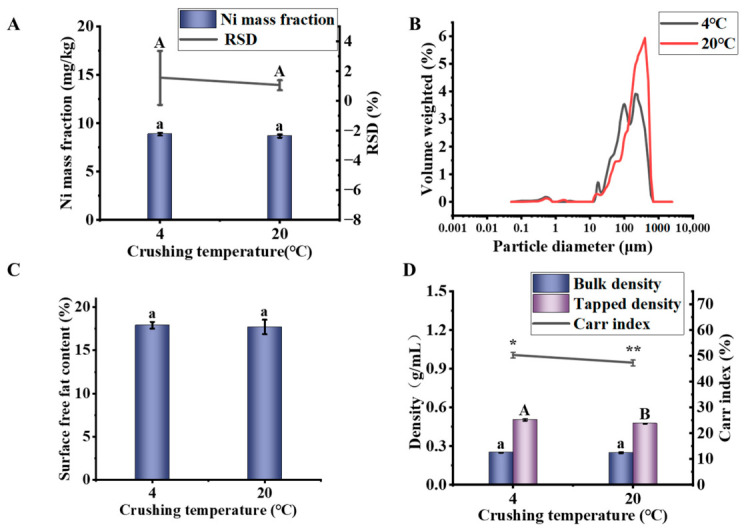
Effects of temperature on the properties of soybean powder. (**A**) Ni mass fraction and its relative standard deviation (RSD). (**B**) Particle size distribution. (**C**) Surface free fat content. (**D**) Particle differentiation characteristics. Different lowercase letters indicate significant differences (*p* < 0.05). Different capital letters indicate significant differences (*p* < 0.05), which are distinguished from lowercase letters. “*, **” for Carr index serve the same purpose, indicating significant differences at *p* < 0.05, respectively, and are used only to visually separate this parameter from the density data.

As shown in Figure 5A, there was no significant difference in Ni content between samples crushed at 4 °C and 20 °C (*p* > 0.05). Error bars represent the standard deviation (SD) of three independent replicate measurements (*n* = 3). The relative standard deviation (RSD, gray line) was calculated from these same replicates, with values below 2% indicating acceptable analytical precision despite the observed variability in absolute Ni content. Notably, the variability was greater at 4 °C. This may be attributed to the semi-solid state of the oil at this temperature [44]. During the crushing process, the oil released from the cells can promote particle re-agglomeration. At the same time, changes in physical properties may lead to selective crushing, further aggravating material inhomogeneity.

To further investigate this mechanism, systematic characterization was conducted. As shown in Figure 5B and Table 7, the span at 20 °C was smaller than that at 4 °C, indicating a narrower and more uniform particle size distribution under room-temperature grinding. This is attributed to plastic deformation and oil-lubricated comminution, which suppress excessive fragmentation. In contrast, at 4 °C, brittle fracture leads to random crack propagation, producing a mixture of fine debris and coarse fragments, thereby increasing the span value and reflecting a broader, less homogeneous size distribution. The differences in span confirm that temperature-induced changes in oil rheology fundamentally alter the breakage mechanism.

As shown in Figure 5C, the surface free fat content at 20 °C was slightly higher than that at 4 °C. However, the surface free fat content showed no significant difference between the two temperatures (*p* > 0.05), because it only reflects the fat on the outermost particle surface and does not capture the internal oil retention that contributes to total oil yield [45].

As shown in Figure 5D, the tapped density (from 0.51 to 0.48 g/mL) and Carr index (from 50.33% to 47.33%) of the powder at 20 °C significantly decreased (*p* < 0.05). This phenomenon indicated that the packing efficiency between particles decreased and interparticle interactions were enhanced [46]. These changes suggest that powder particles may exhibit a more homogeneous packing state at 20 °C, which would reduce local density differences caused by particle segregation. Although flowability was reduced, this relatively stable arrangement contributed to maintaining compositional consistency, which may have a positive effect on homogeneity.

**Figure 6:**
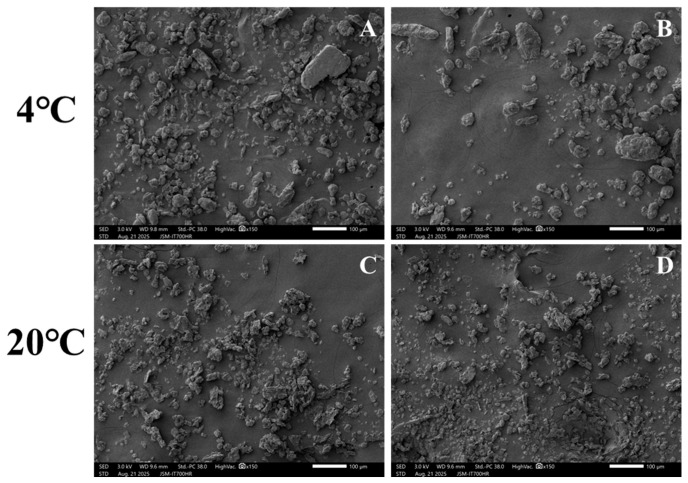
The SEM micrographs of soybean samples at different temperatures. (**A**,**B**): 4 °C; (**C**,**D**): 20 °C.

To further investigate the causes of inhomogeneity, scanning electron microscopy (SEM) was performed. The results are shown in Figure 6. At 4 °C, the powder particles are thicker and more irregular than at 20 ° C. These observations are based on representative SEM images (Figure 6) and are qualitative, although no quantitative morphological analysis was performed. However, it can still be seen that the visualization trend is consistent with expectations; that is, lower temperatures increase brittleness, resulting in wider particle size distributions, which may damage homogeneity [47].

In summary, this study determined that soybeans should be crushed at room temperature. While ensuring crushing efficiency, this temperature also maximizes the homogeneity of the soybean powder. This finding indicates that the selection of temperature needs to balance the properties of each component during the crushing process, rather than simply pursuing the lowest possible temperature.

#### 3.3.3. Rotational Speed

The rotational speed directly affects the powder characteristics by altering the impact energy and velocity [1]. In this study, the relationship between rotational speed and homogeneity was systematically investigated by evaluating the properties of soybean powder processed at different rotational speeds.

**Figure 7:**
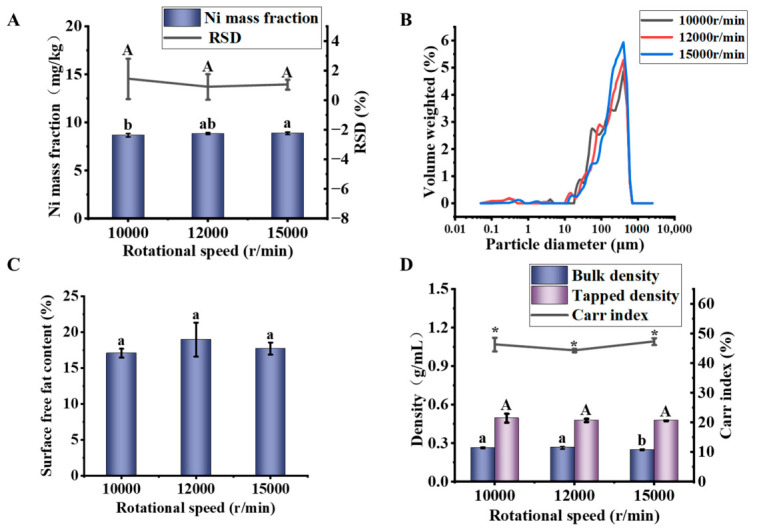
Effects of rotational speed on the properties of soybean powder. (**A**) Ni mass fraction and its relative standard deviation (RSD), (**B**) particle size distribution, (**C**) surface free fat content, (**D**) particle differentiation characteristics. Different lowercase letters indicate significant differences (*p* < 0.05). Different capital letters indicate significant differences (*p* < 0.05), which are distinguished from lowercase letters. “*” for Carr index serve the same purpose, indicating significant differences at *p* < 0.05, respectively, and are used only to visually separate this parameter from the density data.

As shown in Figure 7A, the Ni mass fraction significantly increased with increasing rotational speed (from 8.70 to 8.90 mg/kg), while the Ni-RSD decreased accordingly (from 1.36% to 0.33%). This indicates that increasing rotational speed is an effective method for improving powder homogeneity. This improvement may be attributed to stronger impact and more efficient crushing under high-speed conditions, which produce finer particles and a narrower particle size distribution [48]. Furthermore, the high-speed grinding process inherently promotes vigorous mixing, thereby reducing sampling error

To further analyze the mechanism by which rotational speed affects homogeneity, multi-dimensional characterization was performed. As shown in Figure 7B and Table 8, the particle size distribution revealed that the span continuously decreased as the rotational speed increased from 10,000 r/min to 15,000 r/min. This demonstrates that the highest speed (15,000 r/min) promotes a more concentrated particle size distribution through enhanced impact energy.

As shown in Figure 7C, a nonlinear relationship was observed between surface free fat content and rotational speed. The highest oil yield occurred at 12,000 r/min (18.95%), suggesting that the combined mechanical action and thermal effect promoted the release of the most oil. At a rotational speed of 15,000 r/min, the extremely high speed led to instantaneous fragmentation of the material, resulting in a more uniform particle condition [49]. In other words, the homogeneity of the powder was maintained.

As shown in Figure 7D, at 15,000 r/min, the bulk density (0.25 g/mL, *p* < 0.05) and tapped density (0.47 g/mL, *p* < 0.05) reached their lowest values. These variations in physical properties indicate that increasing rotational speed can improve both homogeneity and structural stability. The change in tapped density directly reflected the improvement in particle packing structure, which is closely related to achieving a more concentrated particle size distribution. The variation in flowability suggests that the risk of segregation was slight. The decrease in bulk density may be associated with reduced particle agglomeration.

In summary, this study identified 15,000 rpm as the best option among the rotational speeds tested. This rotational speed achieves a balance between particle size distribution and compositional homogeneity, thereby maximizing the homogeneity of the soybean powder.

#### 3.3.4. Correlation Analysis

**Figure 8:**
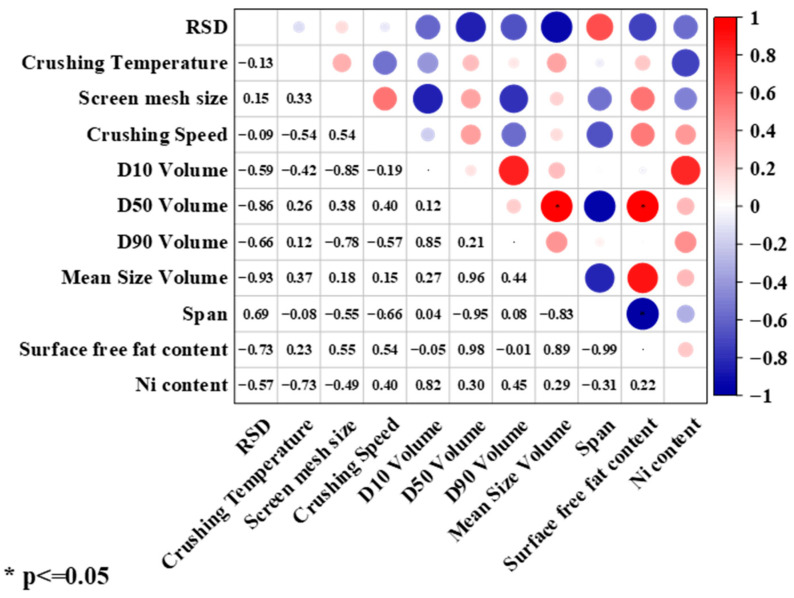
Correlation analysis between crushing process parameters and relative standard deviation (RSD) of Ni mass fraction. The size of each circle is proportional to the absolute value of the correlation coefficient, with larger circles indicating stronger correlations. Color represents correlation direction (red = positive, blue = negative).

As shown in Table S6, Pearson correlation analysis can be performed on the data after normal distribution test. In order to systematically evaluate the effect of process parameters on homogeneity, Pearson correlation analysis was used to explore the correlation between each parameter and Ni-RSD. The results are shown in Figure 8, Tables S7–S9. Multiple linear regression yielded an R^2^ of 1.000 for span with a coefficient of 0.913; however, the ANOVA showed 0 residual degrees of freedom, indicating severe overfitting. Therefore, theregression estimates are unreliable. Despite the non-significant *p*-value, we still use the correlation coefficient for descriptive characterization. Among the various parameters examined, the span showed a positive correlation with homogeneity, Ni-RSD (r = 0.69, r^2^ = 0.48, *p* = 0.127), indicating that a broader particle size distribution tends to be associated with greater elemental inhomogeneity. Notably, the characteristic particle size parameters (D_10_, D_50_, and mean size) were negatively correlated with RSD. This finding reveals a mechanistic insight: process parameters influence homogeneity, primarily by affecting the range of the particle size distribution (span), rather than through direct control of specific particle size thresholds. In other words, simply targeting specific particle size cutoffs is insufficient to ensure homogeneity; instead, controlling the homogeneity of the particle size distribution is a more relevant consideration for process optimization. Based on this mechanistic understanding, the span may serve as a practical indicator for assessing the potential risk of inhomogeneity during the preparation of soybean reference materials, thereby helping to guide milling process adjustments before full chemical characterization.

### 3.4. Homogeneity Study

#### 3.4.1. Homogeneity Study

The soybean powder was prepared for homogeneity assessment. The obtained data were evaluated using analysis of variance (ANOVA). As shown in Table 9, the calculated F-value (1.52) was less than the critical F-value (3.35), and the *p*-value was 0.24 (>0.05), indicating sufficient within-bottle homogeneity. Between-bottle homogeneity was similarly evaluated using ANOVA (Table 10). The calculated F-value (0.89) was less than the critical F-value (2.66), and the *p*-value was 0.53 (>0.05), demonstrating adequate between-bottle homogeneity [25]. Moreover, according to Formulas (1) and (2), the relative uncertainty associated with homogeneity was calculated to be 1.00% (Table 11).

#### 3.4.2. Comparison of Grinding Conditions

For comparison, the same homogeneity test was performed on soybean flour prepared without parameter optimization. The within-bottle ANOVA results (Table 12) showed that the calculated F-value (4.40) exceeded the critical F-value (3.35), with a *p*-value of 0.022 (<0.05), indicating significant within-bottle inhomogeneity. Between-bottle homogeneity was similarly evaluated using ANOVA (Table 13). The calculated F-value (0.69) was less than the critical F-value (2.66), and the *p*-value was 0.68 (>0.05), demonstrating sufficient between-bottle homogeneity. In this case, the between-bottle uncertainty (ubb) was 0.11 mg/kg, corresponding to a relative homogeneity uncertainty of 1.24% (Table 14).

In contrast, under the optimized milling conditions, the between-bottle uncertainty (ubb) was 0.089 mg/kg, with a corresponding homogeneity uncertainty of 1.00% (Table 11). The lower ubb and homogeneity uncertainty achieved under optimized conditions confirm the improvement in between-bottle homogeneity. Notably, the unoptimized material exhibited significant within-bottle inhomogeneity, rendering it unsuitable for use as a reference material, whereas the optimized material met the required homogeneity criteria.

#### 3.4.3. Comparison of Studies

When compared with related studies (Table 15), the uncertainty of homogeneity for elements in pumpkin seed powder ranged from 5% to 23.6% and, in corn powder, from 1.7% to 6.5%. In contrast, the relative uncertainty for Ni in the soybean matrix obtained in this study was only 1.00% (0.089 mg/kg), which is lower than the typical values reported for plant-based reference materials. These results demonstrate the substantial technical advantage of the optimized preparation method in improving homogeneity.

## 4. Conclusions

This study reveals the mechanistic link between the physical properties of soybean tissues and the resulting elemental homogeneity in powder, using Ni as a case study. We demonstrate that the inherent differences in hardness and Ni mass fraction between the seed coat and embryo lead to tissue-specific segregation during milling, which is reflected in both the particle size distribution and the corresponding Ni distribution. Based on this mechanistic understanding, the span of the particle size distribution is proposed as a rapid screening tool to assess the risk of inhomogeneity and to guide the optimization of grinding conditions. This approach does not aim to replace traditional chemical verification by ICP-MS, but rather to reduce the experimental burden by enabling preliminary screening and process control. The findings are particularly relevant for Ni analysis in soybeans and may be extended to other high-fat beans where compositional inhomogeneity is dominated by the differential grindability of tissue components. Further interlaboratory validation is needed to confirm the applicability of this approach to other elements and matrices.

## Figures and Tables

**Table 1:** Microwave digestion procedure.

Step	Hold Time (min)	Temperature (°C)
1	3	100
2	3	120
3	3	150
4	10	180
5	20	190

**Table 2:** Instrument parameters (ICP-MS).

Method Parameters	Value
Power	1550 W
Plasma flow	15.0 L/min
Auxiliary flow	0.9 L/min
Nebulizer flow	0.99 L/min
Pump rate (rps)	0.10 rps

**Table 3:** Mass proportion of each component of soybean seed.

Soybean	Weight (g)	Proportion (%)
Cotyledon	21.13	91.31
Seed coat	1.58	6.83
Germ, radicle, and hypocotyl	0.43	1.86

**Table 4:** Particle size distribution of soybean powder subjected to two milling processes.

Name	D_10_ (µm)	D_50_ (µm)	D_90_ (µm)	Mean Size (µm)	Span
Centrifugal impact milling	42.01	171.92	418.69	223.86	2.19
Hammer cyclone milling	47.44	197.28	410.42	234.94	1.86

**Table 5:** Particle size distribution of soybean powder obtained with different sieve sizes.

Sieve Sizes (mm)	D_10_ (μm)	D_50_ (µm)	D_90_ (µm)	Mean Size (µm)	Span
0.2	33.07	94.52	294.27	139.14	2.74
0.5	40.74	139.36	374.76	193.24	2.40
0.75	40.64	159.08	405.42	214.78	2.29
1.0	43.57	185.36	411.25	229.88	1.98
1.5	49.11	210.93	430.40	250.95	1.81

**Table 6:** Particle size distribution of soybean powder obtained with different mesh numbers.

Mesh Numbers (mm)	D_10_ (µm)	D_50_ (µm)	D_90_ (µm)	Mean Size (µm)	Span
0.3	25.98	171.11	360.13	209.24	1.93
0.45	47.44	197.28	410.42	234.94	1.86

**Table 7:** Particle size distribution of soybean powder subjected to different crushing temperatures.

Temperatures	D_10_ (µm)	D_50_ (µm)	D_90_ (µm)	Mean Size (µm)	Span
20 °C	47.44	197.28	410.42	234.94	1.86
4 °C	44.41	152.64	378.02	197.67	2.20

**Table 8:** Particle size distribution of soybean powder subjected to different rotational speeds.

Rotational Speeds	D_10_ (µm)	D_50_ (µm)	D_90_ (µm)	Mean Size (µm)	Span
10,000 r/min	35.55	137.06	382.63	191.60	2.56
12,000 r/min	47.52	180.22	403.75	223.25	1.98
15,000 r/min	47.44	197.28	410.42	234.94	1.86

**Table 9:** ANOVA results for the within-bottle homogeneity study (optimized conditions).

Source of Difference	Mean Square	F	*p*-Value	F Crit
Between-bottle	0.10	1.52	0.24	3.35
Within-bottle	0.067			
Total				0.059

**Table 10:** ANOVA results for the between-bottle homogeneity study (optimized conditions).

Source of Difference	Mean Square	F	*p*-Value	F Crit
Between-bottle	0.033	0.89	0.53	2.66
Within-bottle	0.037			
Total				0.067

**Table 11:** ANOVA results for the homogeneity study (optimized conditions).

Source of Difference	Value
Total u_bb_ (mg/kg)	0.089
Mass fraction (mg/kg)	8.89
Homogeneity uncertainty (%)	1.00

**Table 12:** ANOVA results for the within-bottle homogeneity study (common conditions).

Source of Difference	Mean Square	F	*p*-Value	F Crit
Between-bottle	0.081	4.40	0.022	3.35
Within-bottle	0.018			
Total				0.079

**Table 13:** ANOVA results for the between-bottle homogeneity study (common conditions).

Source of Difference	Mean Square	F	*p*-Value	F Crit
Between-bottle	0.033	0.69	0.68	2.66
Within-bottle	0.047			
Total				0.074

**Table 14:** ANOVA results for the homogeneity study (common conditions).

Source of Difference	Value
Total ubb (mg/kg)	0.11
Mass fraction (mg/kg)	8.89
Homogeneity uncertainty (%)	1.24

**Table 15:** Research summary of uniformity uncertainties of inorganic elements in different matrix reference materials.

Material	Element	Between-Bottle Homogeneity	Within-Bottle Homogeneity	Homogeneity Uncertainty	Source of Literature
Pumpkin seed flour	K, Mg, P, Zn, Cu, Fe, Mn, Ca	A random sampling method was used to randomly select 8 bottles from 80 bottles as a representative subset.	1. One bottle was randomly selected from the whole batch of samples. 2. Ten sub-samples were randomly selected from the bottle. 3. Under repetitive conditions, the 10 sub-samples were measured.	5–23.6%	[25]
Corn	Ca, K, Mg, P, Zn, Cu, Fe, Mn, Mo	A random sampling method was used to randomly select 20 bottles from 100 bottles as a representative subset.	1. One bottle was randomly selected from the whole batch of samples. 2. Three sub-samples were independently extracted from the bottle. 3. Under repetitive conditions, the three sub-samples were measured.	1.7–6.5%	[50]

## Data Availability

The original contributions presented in the study are included in the article/Supplementary Materials, further inquiries can be directed to the corresponding authors.

## Supplementary Materials

The following supporting information can be downloaded at: https://www.mdpi.com/article/10.3390/foods15091513/s1. Figure S1: The linear relationship of standard curve of Ni.; Table S1: Detection limit and quantitative limit of Ni; Table S2: Matrix solution matching standard curve summary and comparison; Table S3: Recovery of Ni in soybean (n = 3); Table S4: Reproducibility of methods; Table S5: Instrument repeatability investigation; Table S6: Normal distribution test results; Table S7: Correlation analysis results; Table S8: Summary of goodness of fit for the model; Table S9: Model analysis of variance (ANOVA) results.
