# Supplementary material for: Homogeneity Guarantee of Nickel Reference Material in Soybean Matrix: Influence Mechanism of Particle Size Distribution
Source: Foods. 2026 Apr 27;15(9):1513. doi: 10.3390/foods15091513 (PMC13164219; doi:10.3390/foods15091513)
Supplement: Supplementary file 1 [file foods-15-01513-s001.zip › foods-4247391-supplementary.pdf]

Supplementary material for

Homogeneity guarantee of nickel reference material in soybean matrix : Influence mechanism of particle size distribution

Nuojia Wang [1,+], Zengwang Guo [1,+], Yanxiang Wu [2], Jin Ye [2], Lin Zhu [2], Yue Wang [2], Zhongjiang Wang [1,*], Songxue Wang [2,*] and Minghui Zhou [2]

[1] College of Food Science, Northeast Agricultural University, Harbin, Heilongjiang, 150030, China

[2] Academy of National Food and Strategic Reserves Administration, Beijing 100037, China

*Correspondence: Zhongjiang Wang, Songxue Wang

Email addresses: wzjname@126.com (Z.J Wang), wsx@ags.ac.cn (S.X Wang),

1.Method validation

1.1 Linearity, Limit of Detection(LOD), and Limit of Quantification(LOQ)

The calibration curve was prepared by diluting an appropriate amount of Ni standard solution with 5% HNO_3. The curve was constructed with the response value of the target analyte as the vertical axis and the concentration as the horizontal axis.

The limit of detection (LOD) was determined by analyzing the soybean matrix blank sample 11 times under the same operating conditions as the actual sample measurement. The standard deviation (σ) of the blank measurements was calculated, and the LOD for Ni was calculated as $3\sigma \times k^{-1}$, where k is the slope of the Ni calibration curve.

The limit of quantification (LOQ) was defined as three times the LOD (LOQ=3 × LOD).

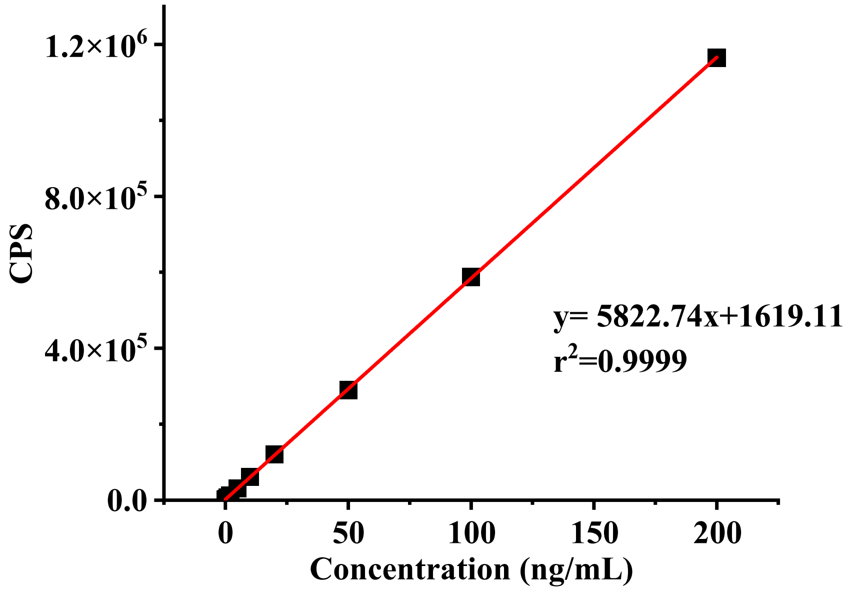

Figure S1. The linear relationship of standard curve of Ni.

Table S1. Detection limit and quantitative limit of Ni.

LOD/LOQ	
Average（mg/kg）	0.00011
SD（mg/kg）	0.03
k	5822.74
LOD（mg/kg）	1.55×10^{-5}
LOQ（mg/kg）	4.64×10^{-5}

1.2 Matrix Effects

At the same concentration, matrix-matched standard solutions and solvent standard solutions were used to calculate the matrix effect. The matrix effect of the method was evaluated based on the ratio of the slope of the matrix-matched calibration curve to the slope of the neat solvent calibration curve. A slope ratio close to 1.0 indicates that the matrix effect is weak and negligible, while a slope ratio between 0.8 and 1.2 is considered acceptable. The calculation formula is shown below. When the slope ratio is greater than 1, the matrix effect is

positive, indicating signal enhancement. When the slope ratio is less than 1, the matrix effect is negative, indicating signal suppression.

Table S2. Matrix solution matching for standard curve summary and comparison.

Standard Curve			
Pure standard solution	$y=25567x+1523.4; R^2=1$		
Soybean matrix matching standard solution	$y=25708x+1523.4; R^2=1$	The average slope of the matching matrix: 25784.25	Matrix effects：1.0085
	$y=25848x+1523.4; R^2=1$		
	$y=25784x+1523.4; R^2=1$		
	$y=25797x+1523.4; R^2=1$		

1.3 Accuracy

Recovery experiment: The soybean sample was divided into four equal portions. Three of the four portions were spiked with Ni at low, medium, and high concentrations, respectively. Recovery was calculated based on the spiked concentration and the measured concentration after spiking to evaluate the accuracy of the method.

Table S3. Recovery of Ni in soybean (n=3).

	**Concentration（mg/kg）**	**Recovery（%）**	**RSD（%）**
	6	95.72	1.16
Ni	12	101.20	1.90
	36	100.31	3.79

1.4 Precision

Method repeatability: A real soybean sample was independently tested seven times, and the mean value and standard deviation were calculated.

Instrument repeatability: The same soybean sample was measured consecutively seven times on the instrument, and the mean value and standard deviation were calculated.

Table S4. Reproducibility of methods.

**Method Repeatability Investigation**	
Average（mg/kg）	10.39
SD（mg/kg）	0.17
CV：%	1.64

Table S5. Instrument repeatability investigation.

**Instrument Repeatability Investigation**	
Average（mg/kg）	10.39
SD（mg/kg）	0.05
CV：%	0.43

1.5 Conclusions

Under the conditions of this method, the matrix effect was 1.0085, the recovery rate was between 95.72 % and 101.20 %, the detection limit was 1.55×10^{-5} mg/kg, the limit of quantitation

was 4.64×10^{-5} mg/kg, and the relative standard deviation RSD≤1.64 %.

2. Correlation Analysis

2.1 Normal Distribution Test

Table S6. Normal distribution test results.

Normal Distribution Test						
	Kolmogorov–Smirnova			Shapiro–Wilk		
	Statistic	df	Significance	Statistic	df	Significance
RSD	0.283	5	0.200*	0.905	5	0.440

*. This is a lower bound of the true significance.

[a]. Lilliefors significance correction

2.2 Correlation Analysis

Table S7. Correlation analysis results.

Correlation Analysis			
		RSD	SPAN
RSD	Pearson correlation	1	0.69
	Significance		0.127
	n	5	5
Span	Pearson correlation	0.69	1
	Significance	0.127	
	n	5	5

2.3 Multiple Regression

Table S8. Summary of goodness of fit for the model.

Model Summary[b]					
Model	R	R^2	Adjusted R^2	Std. Error of the Estimate	Durbin–Watson
1	1.000[a]	1.000			0.273

[a]. Predictors: (constant), span, temperature, mesh, speed.

[b]. Dependent Variable： RSD.

Table S9. Model analysis of variance (ANOVA) results.

ANOVA[a]						
Model		Sum of Squares	df	Mean Square	F	Significance
1	Regression	0.532	4	0.133		[b]
	Residual	0.000	0			
	Total	0.532	4			

[a]. Dependent Variable： RSD.

[b]. Predictors: (constant), span, temperature, mesh, speed.
